# A Multidimensional Questionnaire to Measure Career Satisfaction of Physicians: Validation of the Polish Version of the 4CornerSAT

**DOI:** 10.3390/ijerph17031033

**Published:** 2020-02-06

**Authors:** Juan Nicolás Peña-Sánchez, Alicja Domagała, Katarzyna Dubas-Jakóbczyk, Maciej Polak

**Affiliations:** 1 Department of Community Health and Epidemiology, College of Medicine, University of Saskatchewan, Saskatoon, SK S7N 5E5, Canada; 2 Department of Health Policy and Management, Institute of Public Health, Faculty of Health Sciences, Jagiellonian University Medical College, 31-531 Krakow, Poland; 3 Department of Health Economics and Social Security, Institute of Public Health, Faculty of Health Sciences, Jagiellonian University Medical College, 31-531 Krakow, Poland; katarzyna.dubas@uj.edu.pl; 4 Chair of Epidemiology and Population Studies, Institute of Public Health, Faculty of Health Sciences, Jagiellonian University Medical College, 31-531 Krakow, Poland; maciej.1.polak@uj.edu.pl

**Keywords:** job satisfaction, career, physicians, questionnaire, validation, validity, reliability

## Abstract

To study physicians’ satisfaction with a multidimensional approach, the 4CornerSAT questionnaire to measure the career satisfaction of physicians was conceptualized in English and later adapted into Polish. In this study, we aimed to test the reliability and validity of the adapted 4CornerSAT questionnaire in Poland and confirm its the tetra-dimensional structure. In 2018, physicians working in 15 Polish hospitals were invited to participate in a survey that included the Polish 4CornerSAT. We evaluated the questionnaire’s reliability by computing Cronbach’s alpha coefficients. We also computed a Pearson correlation coefficient between the reported global item of satisfaction and the standardized level of career satisfaction. A confirmatory factorial analysis (CFA) tested the tetra-dimensional structure of the questionnaire in Polish. In total, 1003 physicians participated in this study. The questionnaire’s internal consistency and concurrent validity were optimal. In the CFA, good model fit indicators were observed. In conclusion, the Polish version of the 4CornerSAT demonstrated good psychometric properties. The adapted questionnaire has evidence of its validity and reliability in Poland to be used in further studies and to monitor physicians’ wellness as a health care system indicator. Our approach to adapt and validate this questionnaire could be replicated in other settings.

## 1. Background

The satisfaction of physicians, a positive measure of physicians’ wellness, is a critical indicator of the performance of health care systems, and one that requires the attention of medical practitioners and health care decision-makers [1]. Researchers have used a wide variety of methodological approaches to measure the satisfaction of physicians [2,3]. Indeed, a recent literature review identified more than 40 different types of questioners used among 61 European studies of physicians’ satisfaction [3]. Typically, the concept of job satisfaction has been studied and measured, which could be understood as the individual’s judgment of the job as a whole (e.g., work duties, payment, relationships with supervisors and co-workers, advancements, etc.) and own needs, wants, and expectations [4]. Nevertheless, there is not a standard approach for studying job satisfaction and instruments to measure this concept have validity and reliability limitations [5]. In fact, complex and multiple factors influence job satisfaction of physicians that need to be considered [1,6].

Medical practitioners need to feel challenged and engaged with their job [1,6,7]. The daily work of physicians is heterogeneous, and they require a combination of the intellectually challenging, emotionally satisfying, and socially beneficial components to be satisfied when delivering health care [1,6,8]. There are multiple motivation theories that consider the factors that drive individuals’ behavior to satisfy their own needs or the cognitive processes of motivation assessing efforts and outcomes at work. Among the needs-theories of motivation, the Maslow’s hierarchy of needs is one of the most influential and known theories [8,9]. This theory considers that individuals have lower and higher levels of needs to be satisfied, where higher-order needs can be fulfilled only after satisfying the lower-order ones [8,9]. However, if higher-level needs are not satisfied, people tend to focus their attention on satisfying lower category needs, a concept known as the simple frustration hypothesis [10]. Alderfer developed an alternative needs theory based on a three-level framework of human needs: existence, relatedness, and growth, known as the ERG theory [10]. The McClelland human motivation is another theory that emphasized that a person acquires a specific need over time based on individual experiences; a theory that classifies motivational needs in achievement, power, affiliation, and avoidance [11]. According to the Herzberg’s two-factor theory, there are two set of factors influencing person’s job satisfaction: intrinsic motivators (increasing satisfaction if adequately applied) and extrinsic hygiene factors (reasons for dissatisfaction if deficient) [12]. Any of these theories could guide the study of satisfaction of physicians; notwithstanding, the Maslow’s framework [13] aligns with the concept that physicians are highly trained professionals who, after satisfying lower-order needs, require satisfaction of higher-order needs, such as professional development, life achievement, career growth, autonomy, responsibility, creativity, self-esteem, and self-actualization [1,14]. This concept is essential in health human resources and health care policy research, acknowledging that physicians need to be intellectually challenged, engaged in daily work, emotionally satisfied, and socially gratified when providing health care [1,6,7,8].

A four-dimensional questionnaire to study the career satisfaction of physicians was conceptualized in Canada by Lepnurm et al. [14] based on Maslow’s framework [13]. A 16-item questionnaire, known as the 4cornerSAT, focuses on the longitudinal concept of satisfaction with the career rather than on the transversal emphasis of the satisfaction with the job or current work [14,15]. This questionnaire to measure the career satisfaction of physicians has been used among multiple specialties and in different countries [14,15,16,17,18,19,20,21]. In fact, the 4CornerSAT is helping in identifying that physicians appear to be consistently experiencing low levels of personal satisfaction across countries, a dimension that encompasses the lower order needs of physicians [14,18,20,21].

The 4CornerSAT questionnaire was developed in English and French [14] and subsequently adapted into Spanish [18]. The 4CornerSAT Spanish version was validated in Spain [18] and recently piloted in Colombia [20]. In Poland, this questionnaire was adapted in 2011 following a multiphase methodology [19]. Two bilingual physicians translated and adapted the questionnaire, and an interdisciplinary committee of experts evaluated the translated versions, agreed on a unified version of the questionnaire and validated its cultural adaptation to the context of physicians working in Polish hospitals. Finally, the final Polish version of the questionnaire was piloted with physicians confirming its accuracy and clarity [19].

Given that the health care system in Poland is currently facing a health human resources crisis (i.e., shortage of physicians, emigration of the health providers, and increasing workloads) and the need of health care policies to face this problem [21], we considered it quite relevant and timely to provide a validated questionnaire in Polish to measure career satisfaction of physicians with a multidimensional approach. Consequently, we aimed to continue the evaluation of the psychometric properties of the adapted questionnaire to measure the career satisfaction of physicians in Poland. The objectives of this study were to: (1) test the reliability and validity of the adapted 4CornerSAT questionnaire in Poland, and (2) confirm the tetra-dimensional structure of the Polish questionnaire to measure the career satisfaction of physicians.

## 2. Methods

### 2.1. The Questionnaire

We used the Polish version of the 4CornerSAT questionnaire (Table 1) [19]. This questionnaire has four pre-conceptualized dimensions of physicians’ careers satisfaction [14,19]:

- Personal (i.e., the balance between work and personal life, ability to control own work schedule, and planning of the career advancements)
- Professional (i.e., relationships with health care managers and nurses, payments, and authority of his/her clinical decisions)
- Performance (i.e., access resources to treat patients, meeting patient’s needs, ability to keep medical knowledge updated, and role in organizing prophylactic programs for patients)
- Inherent (i.e., relationships with patients and other physicians, case mix practice, and career development).

The personal and professional dimensions were designed to address Maslow’s lower needs of motivation, while the performance and inherent ones focused on the higher-order needs [1].

After the translation and cultural adaptation to the Polish context of this questionnaire [19], two Polish health care management researchers and experts in the field reviewed the adapted questionnaire in 2018. Given the current Polish context, they recommended considering and testing a new item to evaluate the concept of satisfaction with the interactions and relationships of physicians with their direct supervisors (see item #10 in Table 1). This new item should be tested as part of the professional dimension of satisfaction. As a result, the Polish measure of career satisfaction that we used in this study had 17 items plus a global item. Three dimensions of career satisfaction (personal, performance, and inherent) had four items each and the professional dimension included five items (see Table 1). Before data gathering, the 17-item questionnaire was piloted with five practicing physicians and five experts in the field, corroborating the questionnaire’s accuracy and clarity and making final wording adjustments to the Polish version of the 4CornerSAT. A copy of the Polish and English version of the questionnaire is available in the online Supplementary file.

The questionnaire asked participants to rate “how satisfied are you with” each of the 17 items and the global item on a 6-point Likert scale: i.e., (1) very dissatisfied, (2) dissatisfied, (3) somewhat dissatisfied, (4) somewhat satisfied, (5) satisfied, and (6) very satisfied. We computed standardized levels of career satisfaction by adding scored levels per item and dividing by the 17, producing levels of satisfaction from 1.00 to 6.00. Standardized levels of satisfaction were also computed for each of the four dimensions.

### 2.2. Sample

All physicians working in 15 Polish hospitals were invited to participate in a survey between March and June of 2018 [21]. Participants completed the questionnaire online, with the option to participate on paper if they experienced problems completing the online version. The 15 hospitals were a convenience sample of institutions selected as they were located in 12 different geographical areas of the country, included public and private organizations (12 and 3, respectively). In addition, this sample included a good representation of general, specialist, and university hospitals (7, 5, and 3 institutions, respectively).

The online survey included the 17 items of the Polish career satisfaction questionnaire and sociodemographic questions. Non-respondents received multiple reminders of the online survey following the Dillman method [22]. Each communication stated the scientific nature of the study and that participation was voluntary, as well as the anonymity and confidentiality of the survey. No incentives were offered to participate.

### 2.3. Statistical Analysis

We evaluated the reliability of the questionnaire and each of its four dimensions using Cronbach’s alpha coefficients of internal consistency. The internal consistency of the questionnaire and the professional dimension was tested with and without the new item suggested by the Polish experts. Moreover, we computed a Pearson correlation coefficient between the reported global item of satisfaction and the standardized level of career satisfaction.

To determine the relationships among variables, inter-correlation of the items with Pearson correlations was evaluated. To test sample adequacy and conditions for completing a factor analysis, the Kaiser–Meyer–Olkin and Bartlett sphericity tests were measured. Then, we ran a confirmatory factorial analysis (CFA) to evaluate the tetra-dimensional structure of the questionnaire (i.e., personal, occupational, performance, and inherent satisfaction) defined a priori. We also assessed the appropriateness of including the new item (i.e., Q10) for the Polish context.

We used the Root Mean Square Error of Approximation (RMSEA), Comparative Fit Index (CFI), Normed Fit Index (NFI), Tucker Lewis Index (TLI), and Incremental Fit Index (IFI) as measures of goodness-of-model fit. We considered adequate fit indicators if the RMSEA was less than 0.08, and the CFI, NFI, TLI, and IFI were equal or higher than 0.95 [23,24].

All analyses were completed in R 3.5.3, using the *lavaan* (*v0.6-4*) package. The data were gathered within the project “Career satisfaction of physicians in Poland: comparing levels and associated factors with other European countries in the context of the migration problem” and its methodology had the ethical approval of the Bioethical Committee of the Jagiellonian University (No. 122.6120.290.2016).

## 3. Results

In total, 1035 physicians participated in the survey (response rate of 38%). Data from 32 participants was excluded due to missing information in one or more questionnaire items. Thus, we included data from 1003 physicians in this study. The mean age of the physicians was 43.4 (SD = 11.76) years old, 518 (52%) of participants were males, 485 were females (48%), and 662 (66.3%) of them had a permanent job agreement.

Table 1 presents the descriptive statistics (i.e., mean, standard deviation [SD], median, skewness, and kurtosis) of the 17 items of the questionnaire to measure the career satisfaction of physicians, as well as the statistics for the global item of satisfaction.

The mean standardized level of career satisfaction for the 16-item and 17-item versions were, respectively, 4.02 (SD = 0.70) and 4.06 (SD = 0.67). A strong correlation was observed between the 16-item standardized level of career satisfaction and the global item of satisfaction *r* = 0.79 (*p* < 0.001). A similar level of correlation was observed between the 17-item standardized level of career satisfaction and the global item, *r* = 0.78 (*p* < 0.001).

Per dimension, the mean levels of satisfaction were 3.68 (SD = 0.98) for the personal, 4.18 (SD = 0.77) for the professional (4.05, SD = 0.79, for the 4-item professional scale excluding the new item), 3.97 (SD = 0.86) for the performance, and 4.37 (SD = 0.66) for the inherent. The four dimensions demonstrated positive and statistically significant interdimensional correlations (*r* ≥ 0.76, *p* < 0.001). Table 2 presents the corrected item-total correlation and inter-item correlation indexes.

The Polish 4CorserSAT questionnaire had optimal internal consistency reliability, α = 0.901 for the 16-item questionnaire and α = 0.902 for the questionnaire including the new item. Very good internal reliability levels were observed in the personal (α = 0.82), professional (α = 0.77 for the four-item scale and α = 0.80 for the five-item scale), performance, (α = 0.83), and inherent (α = 0.81) dimensions of career satisfaction.

**Figure 1:**
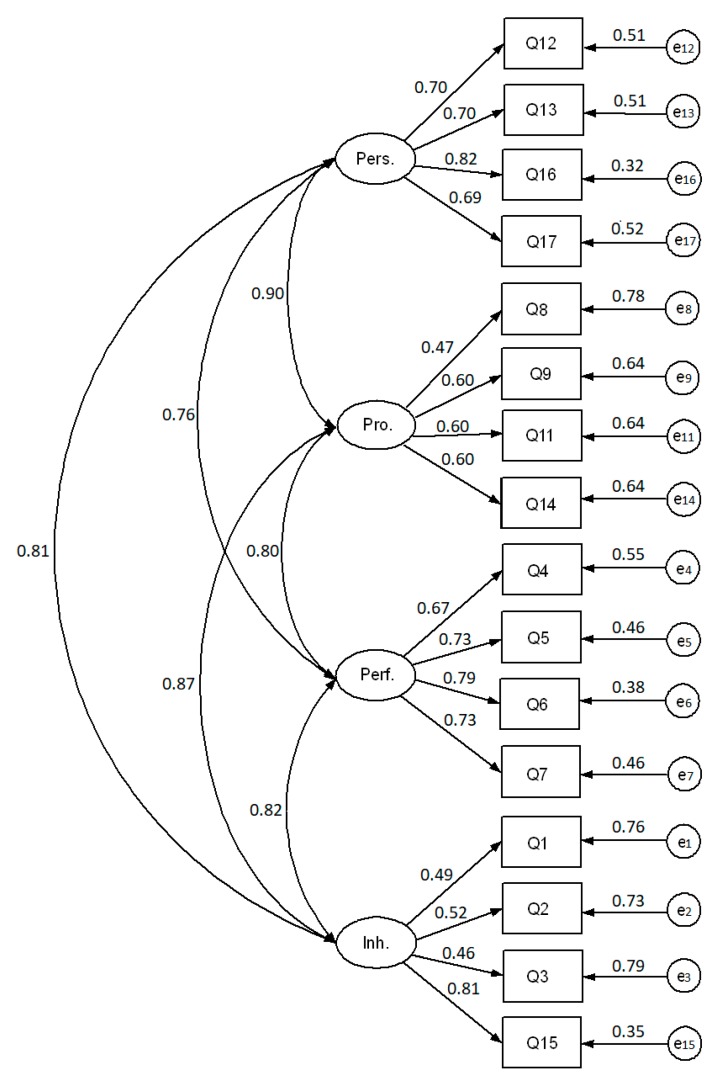
Confirmatory factor analysis estimates of the 16-item questionnaire in Polish to measure the career satisfaction of physicians. The dimensions of career satisfaction are personal (pers.), professional (pro.), performance (perf.), and inherent (inh.). This analysis was completed with the original 16 items of the 4CornerSAT in Polish.

**Figure 2:**
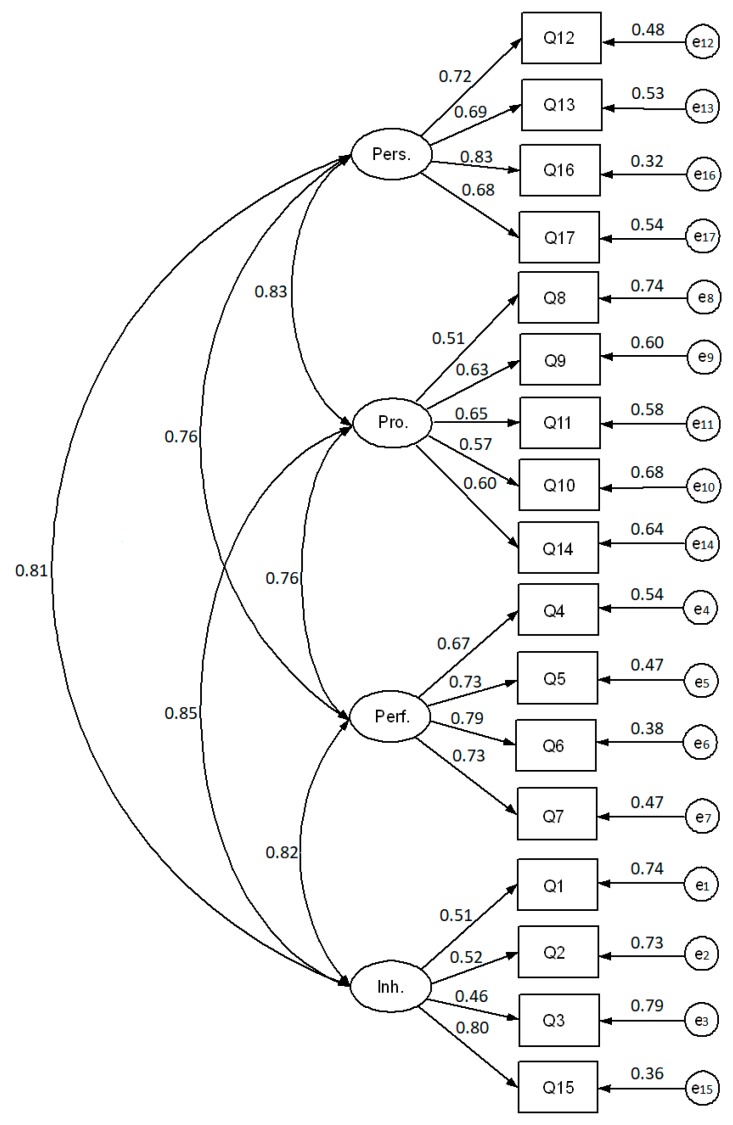
Confirmatory factor analysis estimates of the 17-item questionnaire in Polish to measure the career satisfaction of physicians. The dimensions of career satisfaction are personal (pers.), professional (pro.), performance (perf.), and inherent (inh.). This analysis was completed with 17 items, the original 16 items of the 4CornerSAT in Polish plus the new item evaluating relationships with direct supervisors (i.e., Q10).

The Kaiser–Meyer–Olkin test (KMO = 0.881) and Bartlett sphericity test (χ^2^ = 7,189.4, *p* < 0.001) showed sample adequacy and suitability for completing a factorial analysis. The model fit indicators of CFA (RMSEA = 0.067 [90%CI 0.062–0.073], CFI = 0.96, NFI = 0.95, TLI = 0.96, and IFI = 0.96) demonstrated a very good model fit of the four-dimensional structure of the 16-items questionnaire in Polish. These results were quite similar in the 17-item questionnaire that included the new item (RMSEA = 0.075 [90%CI 0.072−0.078], CFI = 0.95, NFI = 0.95, TLI = 0.94, and IFI = 0.96). Given these results, we did not consider necessary to eliminate any items or add a covariance to the model. Figure 1 and Figure 2 present the results of these CFAs with the standardized values.

## 4. Discussion

The Polish version of the 4CornerSAT has demonstrated optimal psychometric properties. Given that the Cronbach’s alpha coefficient was over 0.9 [25], we could infer that the internal consistency of this questionnaire in Poland was adequate. Regarding the concurrent validity [25] of the Polish 4CornerSAT, a strong correlation between the standardized level of career satisfaction and the global item of satisfaction was demonstrated. In fact, a better correlation was observed between the 17-item standardized satisfaction and the global item. Furthermore, good model fit indicators were observed in the CFA [23,24], confirming the construct validity of the questionnaire and endorsing the tetra-dimensional structure of the questionnaire to measure the career satisfaction of physicians in Poland. Similar model fit indicators were observed when including the item to assess the relationship with the direct supervisors. The internal consistency of each dimension was also adequate. We want to highlight that we observed better internal reliability in the professional dimension when including the item assessing the relationship with the direct supervisors. These psychometric characteristics, along with the methodological rigour to adapt the questionnaire into the Polish context [19], demonstrate the reliability and validly of the 4CornerSAT questionnaire in Poland.

Previous studies in Poland applied two different approaches to assess the satisfaction of physicians. Gaszynska et al. [26] and Lewtak et al. [27] measured physicians’ satisfaction using a modified version of the questionnaire of Bovier and collaborators [28], a 17-item work satisfaction questionnaire developed in French with five dimensions (i.e., patient care, work-related burden, income-prestige, personal rewards, and relations with colleagues). Despite there was some evidence of the adapted questionnaire’s reliability [26], it was not described how this measure was adapted and validated in the Polish context. Tartas et al. [29,30,31] measured satisfaction with medicine using a self-designed tool based on the Cantril’s Scale method. This measure of career satisfaction used an original approach and had good reliability [29,30,31]; although, this measure did not take into consideration the multifaceted nature of the career satisfaction concept. We are presenting a multidimensional questionnaire that has been adapted into Polish through a rigorous multistage process [19], and that has very good psychometric characteristics among physicians working in Polish hospitals. Moreover, the 4CornerSAT questionnaire is available in four different languages and countries [14,18,19,20,21].

Numerous tools to measure the satisfaction of physicians have been used around the world; this reality reflects the diversity of approaches and, at the same time, a lack of standardization and comparability [2,3,5]. Within this diversity, a few questionnaires have demonstrated high validity and reliability [5]. A lack of validated and reliable psychometric instruments limit study results, as well as their comparability [32]. Also, researchers call for international collaborative studies to better understand the satisfaction of physicians and associated factors [2,33]. Similarly, multifaceted measures of physicians’ satisfaction are needed to understand challenges and define effective actions to improve the wellness of physicians [2]. These kinds of initiatives require valid and equivalent measurements of physicians’ satisfaction across countries to contrast and compare findings. The 4CornerSAT is a multidimensional questionnaire that presents as an option to conduct international studies [14,18,19,20,21]. In addition, we endorse the replication of our adaptation and validation methodology, which has demonstrated its effectiveness in two different contexts [15,19].

We acknowledge the limitations of our study. Despite the adequate response rate and sample size, there is still the potential selection bias of study participants as they volunteered to be part of the study. Also, we were not able to assess other important validity and reliability concepts in the Polish 4CornerSAT questionnaire, such as test-retest reliability, predictive validity, or discriminant validity. The evaluation of these additional psychometric characteristics would require the completion of a survey at different time points and the inclusion of other tools will make the survey longer. Either follow-up surveys or a longer questionnaire could negatively affect the participation rate and, subsequently, the study results. Notwithstanding, the gathered evidence of the questionnaire’s validity and reliability let us recommend the use and further testing of the Polish version of the 4CornerSAT in further studies.

## 5. Conclusions

After the completed multistage adaptation process, the Polish version of the 4CornerSAT questionnaire to measure the career satisfaction of physicians has demonstrated optimal psychometric properties. The tetra-dimensional structure of Polish 4CornerSAT has been also confirmed. The adapted questionnaire has evidence of its validity and reliability in Poland to be used in further studies. In addition, the availability of this questionnaire in multiple languages could facilitate further studies aimed to evaluate the career satisfaction of physicians across countries. This questionnaire has the potential to contribute to monitoring physicians’ wellness as a health care system indicator. We also want to highlight that the used multistage approach to adapt and validate the 4CornerSAT questionnaire into the Polish context could be replicated in other settings within the field of health care providers’ satisfaction, as well as in other fields that require comparable and valid psychometric tools across countries and languages.

## Figures and Tables

**Table 1:** Descriptive statistics items of the 4CornerSAT to measure the career satisfaction of physicians (*n* = 1003); the questionnaire items are organized by dimensions.

	Item/Dimension	Mean (SD)	Median	Skewness	Kurtosis
	**Personal**				
Q12	Your ability to control your work schedule?	4.18 (1.13)	4	−0.69	0.30
Q13	Your work-personal life balance?	3.23 (1.33)	3	0.00	−0.81
Q16	Planning of your career advancements?	3.99 (1.09)	4	−0.65	0.36
Q17	Your ability to maintain satisfying activities in the community (service, culture, church, etc.)?	3.31 (1.32)	3	−0.11	−0.78
	**Professional dimension**				
Q8	Your interactions and relationship with nurses?	4.62 (0.91)	5	−0.65	1.06
Q9	Your interactions and relationship with hospital administration/management?	3.94 (1.21)	4	−0.60	0.01
Q10 *	Your interactions and relationship with your direct supervisor?	4.69 (1.07)	5	−0.91	0.98
Q11	Your authority to get your clinical decisions carried out?	4.52 (0.99)	5	−0.75	0.93
Q14	Your earnings as a physician?	3.12 (1.35)	3	0.00	−0.86
	**Performance dimension**				
Q4	Your success in meeting the needs of your patients?	4.48 (0.94)	5	−0.67	1.05
Q5	Your ability to access resources needed to treat your patients?	3.87 (1.14)	4	−0.47	-0.05
Q6	Your capacity to keep up with advances in your clinical specialty?	3.98 (1.10)	4	−0.53	0.10
Q7	Your role in organizing prophylactic programs for patients?	3.56 (1.09)	4	−0.21	−0.15
	**Inherent dimension**				
Q1	Your interactions and relationship with other physicians?	4.60 (0.88)	5	−0.72	1.56
Q2	The doctor-patient relationships derived from providing patient care?	4.35 (0.83)	4	−0.49	1.21
Q3	The diversity of patients you see (age, types of clinical conditions, etc.)?	4.51 (0.88)	5	−0.56	1.20
Q15	Your career advancement in medicine?	4.04 (1.09)	4	−0.69	0.50
Global item	Taking into account all factors, you assess your medical career as	4.09 (0.96)	4	−0.75	0.83

* New item in the Polish version of the questionnaire.

**Table 2:** Inter-item correlations 4CornerSAT questionnaire to measure the career satisfaction of physicians in Polish.

	Inter-Item Correlation	Q1	Q2	Q3	Q4	Q5	Q6	Q7	Q8	Q9	Q10	Q11	Q12	Q13	Q14	Q15	Q16	Q17
Q1	0.52	1																
Q2	0.52	0.38 *	1															
Q3	0.47	0.34 *	0.48 *	1														
Q4	0.63	0.28 *	0.40 *	0.52 *	1													
Q5	0.67	0.26 *	0.25 *	0.22 *	0.50 *	1												
Q6	0.71	0.32 *	0.32 *	0.33 *	0.51 *	0.69 *	1											
Q7	0.68	0.27 *	0.33 *	0.27 *	0.43 *	0.53 *	0.53 *	1										
Q8	0.52	0.44 *	0.30 *	0.24 *	0.35 *	0.27 *	0.28 *	0.28 *	1									
Q9	0.63	0.32 *	0.26 *	0.15 *	0.28 *	0.43 *	0.36 *	0.47 *	0.35 *	1								
Q10	0.57	0.43 *	0.23 *	0.21 *	0.30 *	0.28 *	0.33 *	0.26 *	0.43 *	0.47 *	1							
Q11	0.64	0.37 *	0.28 *	0.28 *	0.36 *	0.29 *	0.35 *	0.30 *	0.36 *	0.36 *	0.55 *	1						
Q12	0.70	0.35 *	0.27 *	0.21 *	0.34 *	0.39 *	0.35 *	0.40 *	0.34 *	0.43 *	0.47 *	0.68 *	1					
Q13	0.67	0.21 *	0.29 *	0.21 *	0.31 *	0.39 *	0.40 *	0.46 *	0.19 *	0.34 *	0.22 *	0.31 *	0.45 *	1				
Q14	0.61	0.16 *	0.21 *	0.16 *	0.27 *	0.43 *	0.34 *	0.34 *	0.20 *	0.39 *	0.21 *	0.31 *	0.38 *	0.50 *	1			
Q15	0.75	0.31 *	0.31 *	0.34 *	0.43 *	0.42 *	0.56 *	0.43 *	0.31 *	0.35 *	0.37 *	0.46 *	0.48 *	0.43 *	0.46 *	1		
Q16	0.77	0.33 *	0.32 *	0.32 *	0.44 *	0.43 *	0.53 *	0.44 *	0.32 *	0.38 *	0.36 *	0.47 *	0.55 *	0.48 *	0.46 *	0.83 *	1	
Q17	0.66	0.14 *	0.27 *	0.17 *	0.30*	0.39 *	0.42 *	0.46 *	0.16 *	0.35 *	0.16 *	0.25 *	0.39 *	0.73 *	0.47 *	0.53 *	0.57 *	1

* *p* < 0.001.

## Supplementary Materials

The following are available online at https://www.mdpi.com/1660-4601/17/3/1033/s1, Table S1: Polish version of the 4CornerSAT questionnaire, and Table S2: English version of the 4CornerSAT questionnaire.
