# Supplementary material for: A Multidimensional Questionnaire to Measure Career Satisfaction of Physicians: Validation of the Polish Version of the 4CornerSAT
Source: Int J Environ Res Public Health. 2020 Feb 6;17(3):1033. doi: 10.3390/ijerph17031033 (PMC7037848; doi:10.3390/ijerph17031033)
Supplement: Supplementary file 1 [file ijerph-17-01033-s001.pdf]

Pomiar satysfakcji zawodowej lekarzy

Wersja polska (Polish version)*

	W jakim stopniu / Jak bardzo jest Pan/i zadowolony/a z…	Bardzo niezadowolony	Niezadowolony	Raczej niezadowolony	Raczej zadowolony	Zadowolony	Bardzo zadowolony
1	Z relacji z innymi lekarzami	[ ]	[ ]	[ ]	[ ]	[ ]	[ ]
2	Z relacji z pacjentami	[ ]	[ ]	[ ]	[ ]	[ ]	[ ]
3	Z różnorodności pacjentów i ich schorzeń	[ ]	[ ]	[ ]	[ ]	[ ]	[ ]
4	Z możliwości pomocy pacjentom	[ ]	[ ]	[ ]	[ ]	[ ]	[ ]
5	Z dostępu do zasobów i środków niezbędnych do leczenia pacjentów	[ ]	[ ]	[ ]	[ ]	[ ]	[ ]
6	Z możliwości dotrzymywania kroku postępom wiedzy w Pana/i specjalizacji klinicznej	[ ]	[ ]	[ ]	[ ]	[ ]	[ ]
7	Z możliwości prowadzenia działań profilaktycznych wśród swoich pacjentów	[ ]	[ ]	[ ]	[ ]	[ ]	[ ]
8	Z relacji z pielęgniarkami	[ ]	[ ]	[ ]	[ ]	[ ]	[ ]
9	Z relacji z administracją szpitala	[ ]	[ ]	[ ]	[ ]	[ ]	[ ]
10	Z relacji z bezpośrednim przełożonym	[ ]	[ ]	[ ]	[ ]	[ ]	[ ]
11	Z zakresu autonomii w podejmowaniu decyzji klinicznych	[ ]	[ ]	[ ]	[ ]	[ ]	[ ]
12	Ze swobody w planowaniu własnej pracy	[ ]	[ ]	[ ]	[ ]	[ ]	[ ]
13	Z równowagi pomiędzy życiem zawodowym a prywatnym	[ ]	[ ]	[ ]	[ ]	[ ]	[ ]
14	Z poziomu wynagrodzenia	[ ]	[ ]	[ ]	[ ]	[ ]	[ ]
15	Ze swojego rozwoju zawodowego	[ ]	[ ]	[ ]	[ ]	[ ]	[ ]
16	Ze sposobu planowania własnej ścieżki zawodowej	[ ]	[ ]	[ ]	[ ]	[ ]	[ ]
17	Z możliwości utrzymywania aktywności pozazawodowej (np. społecznej i kulturalnej)	[ ]	[ ]	[ ]	[ ]	[ ]	[ ]
	**Biorąc pod uwagę wszystkie czynniki, swoją satysfakcję z wykonywania zawodu lekarza oceniam jako**	[ ]	[ ]	[ ]	[ ]	[ ]	[ ]

* Questionnaire adapted by Peña-Sánchez JN, Domagala A, Górkiewicz M, Targowska M, Oleszczyk M *(Adapting a tool in Poland for the measurement of the physicians' career satisfaction. Problemy Medycyny Rodzinnej [Family Medicine Topics] 2011; 12(1):58-65)* and validated by Peña-Sánchez, J.N., Domagała, A., Dubas-Jakóbczyk, K., Polak M *(A multidimensional questionnaire to measure career satisfaction of physicians: validation of the Polish version of the 4CornerSAT. Int. J. Environ. Res. Public Health, 2020).*

Questionnaire to measure career satisfaction of physicians

English version**

	How satisfied are you with:	Very dissatisfied	Dissatisfied	Somewhat dissatisfied	Somewha t satisfied	Satisfied	Very satisfied
1	Your interactions and relationship with other physicians?	[ ]	[ ]	[ ]	[ ]	[ ]	[ ]
2	The doctor-patient relationships derived from providing patient care?	[ ]	[ ]	[ ]	[ ]	[ ]	[ ]
3	The diversity of patients you see (age, types of clinical conditions, etc)?	[ ]	[ ]	[ ]	[ ]	[ ]	[ ]
4	Your success in meeting the needs of your patients?	[ ]	[ ]	[ ]	[ ]	[ ]	[ ]
5	Your ability to access resources needed to treat your patients?	[ ]	[ ]	[ ]	[ ]	[ ]	[ ]
6	Your capacity to keep up with advances in your clinical speciality?	[ ]	[ ]	[ ]	[ ]	[ ]	[ ]
7	Your role in organizing treatment programs for patients in your community?	[ ]	[ ]	[ ]	[ ]	[ ]	[ ]
8	Your interactions and relationship with nurses?	[ ]	[ ]	[ ]	[ ]	[ ]	[ ]
9	Your interactions and relationship with health care administrators?	[ ]	[ ]	[ ]	[ ]	[ ]	[ ]
10	Your authority to get your clinical decisions carried out?	[ ]	[ ]	[ ]	[ ]	[ ]	[ ]
11	Your ability to control your work schedule?	[ ]	[ ]	[ ]	[ ]	[ ]	[ ]
12	Your ability to keep responsibilities at work from intruding on your personal life?	[ ]	[ ]	[ ]	[ ]	[ ]	[ ]
13	Your earnings as a physician during your medical career?	[ ]	[ ]	[ ]	[ ]	[ ]	[ ]
14	Your Career advancement in medicine?	[ ]	[ ]	[ ]	[ ]	[ ]	[ ]
15	The way your medical practice is managed?	[ ]	[ ]	[ ]	[ ]	[ ]	[ ]
16	Your ability to maintain satisfying activities in the community (service, culture, church, etc)?	[ ]	[ ]	[ ]	[ ]	[ ]	[ ]
17	Your medical career, considering your various roles and responsibilities?	[ ]	[ ]	[ ]	[ ]	[ ]	[ ]

**Questionnaire developed by Lepnurm R, Danielson D, Dobson R, Keegan D *(Cornerstones of career satisfaction in medicine. Can J Psychiatry 2006;51:512-22)*
